# Proceedings of Norfolk District Medical Society

**Published:** 1877-06

**Authors:** Henry A. Martin

**Affiliations:** Chairman


### Proceedings of Norfolk District Medical Society.

Dr. Henry A. Martin presented the following report of a committee of the Norfolk District Medical Society concerning the propriety of payment being demanded for certain certificates now required gratuitously, and under penalties, by the state and municipal governments.

Your committee, appointed to determine and report what measures, if any, may properly be taken with a view to the payment of members of this society for the preparation and signature of certain certificates required by law, begs leave to say that two forms of such certificates are known to it:—

(1.) Certificates or notifications of the existence of disease dangerous to the community by reason of contagion, as small-pox, scarlet fever, and diphtheria.

(2.) Certificates of death and causes of death. It is not considered necessary to recite the statutes which make the return of such certificates obligatory. They are both required by law, disobedience to which involves a considerable penalty.

In regard to certificates of the existence of dangerous contagious diseases, the members of your committee are unanimously of the opinion that such certificates are most properly required, and should be willingly, promply, and accurately made, on the ground of public necessity, as aid may be demanded for the suppression of a riot or the arrest of a criminal, and can be withheld only under the penalty of arrest and punishment as an accessory,—*particeps criminis.* While your committee recommends that this class of certificates should be rendered by the profession as a matter of the highest and most imperative duty, it would insist that the performance of the duty should not entail a pecuniary tax, however slight, upon him who performs it. Proper blanks should be furnished, and also stamped envelopes with printed addresses rendering them valueless for other uses. Your committee is aware that postal cards have lately been, to a limited extent, distributed for this purpose, but is of opinion that there are reasons of considerable force why these notifications should be under cover of an envelope.

The second variety of certificate, that of death and cause of death, your committee considers of very great importance, and fully agrees that such certificates should be made with the greatest care, accuracy, and promptness. For many very important reasons this last is demanded. Such service of physicians is demanded not by public necessity or safety, but for the public good, precisely as that of a juryman or witness, whose services are for the public good and may be imperatively demanded, but must be paid for. There seems to be no reason whatever why such certificates should be required under penalty of a fine, when no remuneration is afforded for the faithful performance of the required service.

That such certificates should be paid for would seem to be admitted by the payment of a certain fee to the undertaker for merely certifying death and burial. There surely is no valid reason why an undertaker should be paid for giving a certificate which requires no skill or knowledge whatever, except perhaps a very limited acquaintence with chirography, and a physician be allowed no remuneration for a similar service, requiring, when properly performed, both skill and special knowledge, and often in no slight degree;—no reason whatever, except the usage of demanding gratuitous service from our profession, usage encouraged and developed by the facility with which the profession of medicine has rendered and does render, in a thousand ways, unpaid service to humanity.

Your committee would recommend that a form of petition be prepared, in such way as may be hereafter decided, asking from the legislature such changes in existing statutes as may enable physicians to obtain a reasonable fee for the preparation and signature of certificates of death and its cause. Your committee also recommends that the secretary be directed to communicate with the secretaries of other district societies throughout the State, which together constitute the Massachusetts Medical Society, such action as may be taken in this matter, with a request for their cooperation, to the end that the petition above recommended may be that of *all* the physicians of Massachusetts in regular standing.

Another variety of certificate, namely, those showing that individuals have been duly vaccinated, has come under the notice of your committee. Such papers are continually called for, sometimes to a degree involving great trouble and loss of time. Physicians are, however, not required to give such certificates by any statute or ordinance. The law requires that every child seeking to enter one of the public schools shall present such a certificate as a prerequisite to admission. The parents or guardians must furnish such certificates; no physician, however, except the city or town physician, where there is such a functionary, is bound to furnish them free of charge. Although in fact an immense proportion of these vaccination certificates are furnished gratuitously by physicians, there is no doubt whatever that they are entitled to a proper fee for such certificates and for the previous skilled examination essential to their proper preparation, as for any other professional labor. It would be very difficult, however, to obtain such remuneration from individuals, and as the rendering of such certificates is eminently for the public good, your committee considers that it should be paid for by the public.

The question of remuneration for certificates of vaccination is one for the consideration, under existing statutes, of the different municipal governments. Your committee is informed that some of these governments within this district have rerognized the propriety of remuneration, and allow a moderate fee for each certificate of vaccination. Your committee recommends that representation be made to each municipal government, by the members of this society resident therein, of the justice and propriety of remuneration for making out certificates of vaccination, and does not doubt that such representation will be favorably considered. It is desirable that there should be uniformity in the fee asked for, and your committee, after careful consideration, would name fifty cents as a moderate and fit sum to be paid for each certificate, either of vaccination or of death and its cause. Your committee is, however, of opinion that such fee or any fee should only be payable by either the state or the municipal government for a careful, accurate, and prompt performance of the service required. In cases in which certificates of due vaccination should be given to children whose bodies do not present the clear characteristic mark or marks which alone can authorize such a certificate being given at all, unless after a most careful and, if necessary, repeated re-vaccination, not only would the authorities be fully justified in withholding remuneration, but also in inflicting an exemplary penalty.

In a word, your committee would most earnestly represent that all certiffcates from physicians should be made out carefully, accurately, fully, and promptly. All these the state has a right to expect from the members of a learned and liberal profession, and in cases where negligence, inaccuracy, ignorance, or delay is found. a perfect right also to withhold remuneration, and in extreme cases to enforce the penal clauses of the statutes. On the other hand, when such service is done with scientific accuracy and in all respects as the law requires, the profession has a right to expect and demand a moderate and reasonable payment for the service rendered to the community.

Henry A. Martin, *Chairman.*

—*Boston Med· and Surg. Journal.*

